# Unraveling the role of macrophages in diabetes: Impaired phagocytic function and therapeutic prospects

**DOI:** 10.1097/MD.0000000000041613

**Published:** 2025-02-21

**Authors:** Bing Rong, Hailun Jiang, Weiming Zhu, Guanhu Yang, Xuancheng Zhou, Zhongxi Lyu, Xiangyi Li, Jieying Zhang

**Affiliations:** a First Teaching Hospital of Tianjin University of Traditional Chinese Medicine, Tianjin, China; b National Clinical Research Center for Chinese Medicine Acupuncture and Moxibustion, Tianjin, China; c Graduate School, Tianjin University of Traditional Chinese Medicine, Tianjin, China; d Department of Specialty Medicine, Ohio University, Athens, OH; e Clinical Medical College, Southwest Medical University, Luzhou, China; f School of Acupuncture & Moxibustion and Tuina, Tianjin University of Traditional Chinese Medicine, Tianjin, China; g Tianjin University of Traditional Chinese Medicine, Tianjin, China.

**Keywords:** autophagy, diabetes, efferocytosis, inflammation, macrophages, phagocytosis

## Abstract

The rising aging population and changing lifestyles have led to a global increase in diabetes and its complications, making it one of the most prevalent diseases worldwide. Chronic inflammation is a key pathogenic feature of diabetes and its complications, yet the precise mechanisms remain unclear, impeding the development of targeted therapies. Recent studies have highlighted the β cell-macrophage crosstalk pathway as a crucial factor in chronic low-grade inflammation and glucose homeostasis imbalance in both type 1 and type 2 diabetes. Furthermore, impaired macrophage phagocytic functions, including pathogen phagocytosis, efferocytosis, and autophagy, play a significant role in diabetes complications. Given their high plasticity, macrophages represent a promising research target. This review summarizes recent findings on macrophage phagocytic dysfunction in diabetes and its complications, and explores emerging therapies targeting macrophage phagocytic function. We also discuss the current challenges in translating basic research to clinical practice, aiming to guide researchers in developing targeted treatments to regulate macrophage status and phagocytic function, thus preventing and treating metabolic inflammatory diseases.

## 1. Introduction

In contemporary society, diabetes stands as one of the most prevalent and serious chronic diseases. According to the International Diabetes Federation, as of 2019, 463 million individuals globally were afflicted with diabetes, reflecting a prevalence rate of 9.3%. Projections indicate that by 2030, this number will rise to 578 million (10.2%), and by 2045,^[1]^ it will reach 700 million (10.9%). Understanding the mechanisms behind diabetes onset and progression is crucial for developing and implementing multi-sectoral response measures to reduce risk factors and formulate targeted therapies, significantly impacting the reduction of diabetes prevalence.

Various animal models of diabetes studied to date have shown islet immune cell infiltration^[2–4]^ and inflammation as key pathogenic features of diabetes. Islets from diabetic patients exhibit elevated concentrations of pro-inflammatory factors, production of various chemokines, and increased levels of macrophages.^[5,6]^ The persistent inflammatory environment can increase fasting blood glucose by regulating the hepatic mevalonate pathway through CYP7A1, which inhibits bile acid biosynthesis.^[7]^ Proinflammatory cytokines tumor necrosis factor alpha (TNF-α) and interleukin-6 (IL-6) also interfere with insulin signal transduction, causing insulin resistance and elevated blood glucose.^[8–11]^ Although the underlying mechanisms of diabetes are still unclear, there is growing evidence that the β cell-macrophage crosstalk pathway may contribute to chronic low-grade inflammation and glucose homeostasis imbalance in Type 1 diabetes mellitus (T1DM) and Type 2 diabetes mellitus (T2DM).^[12–14]^

Macrophages are innate immune cells. They are main source is circulating monocytes, which can be activated by pattern recognition receptors, such as Toll-like receptors (TLRS). The immune response is to the molecular structures on the pathogen-associated molecular patterns (PAMPs) and the damage-associated molecular patterns (DAMPs), a class of substances released into the intercellular or blood circulation after tissue or cell damage.^[15,16]^ It is generally believed that changes in the internal environment can drive macrophages to differentiate into specific subsets of macrophages to perform corresponding functions, such as the ability to release (cytokines, chemokines, proteases) and phagocytose^[17]^ (dead or dying cells in the body, viruses, bacteria, parasites, fungi and foreign body particles) during inflammation.^[18]^ It can also display repair ability after tissue injury (stimulate blood vessel development,^[19]^ promote peripheral nerve regeneration,^[20]^ promote fracture healing.^[21,22]^ In terms of diabetes and its complications, the above characteristics of macrophages play a dual role in protecting and damaging the cells.

In this review, we aim to elucidate the crucial role of macrophage-mediated phagocytosis, a key function of innate immune cells, in the contexts of T1DM and T2DM and their complications. We will discuss the involvement of abnormal phagocytic and efferocytic processes in the initiation and progression of diabetes and its complications, explore the impact of dysfunctional macrophagic autophagy on diabetic complications, and examine potential therapeutic targets involving macrophages. These aspects are addressed to provide a comprehensive perspective and reference for diabetes research.

## 2. Macrophage and its polarization

Macrophages play an indispensable role in the immune system, fulfilling functions such as phagocytosis, tissue repair, and pro- and anti-inflammatory responses.^[2]^ They are the primary effector cells in innate immunity, having the ability to engulf pathogens and cellular debris, secrete cytokines, and mediate immune responses in accordance with environmental cues.

### 2.1. Overview of macrophage functions

Macrophages play a crucial role in immune response and tissue maintenance.^[4]^ Firstly, through phagocytosis, they ingest and degrade pathogens and damaged cells, thus maintaining tissue homeostasis. Then, following tissue injury, they support tissue repair by secreting growth factors and promoting angiogenesis. Next, during infection or injury, macrophages initiate a pro-inflammatory response by releasing cytokines such as TNF-α and IL-6, which recruit immune cells to enhance local inflammation. Finally, they also contribute to anti-inflammatory responses by releasing cytokines like IL-10 and TGF-β, which help alleviate inflammation and promote tissue healing.

### 2.2. Mechanism of polarization

Macrophages polarize into 2 main types, M1 (pro-inflammatory) and M2 (anti-inflammatory), each serving distinct functions. Specifically, M1 macrophages are activated by IFN-γ and lipopolysaccharide (LPS), exhibiting strong pro-inflammatory properties as they release TNF-α and IL-1β to combat pathogens and clear damaged tissues. In contrast, M2 macrophages are stimulated by IL-4 and IL-13 and are primarily involved in anti-inflammatory responses, secreting IL-10 and TGF-β to promote tissue repair and reconstruct the extracellular matrix. Overall, this polarization allows macrophages to adapt dynamically to immune demands, balancing defense and repair, a feature particularly important in managing diabetes and its complications.

## 3. Abnormal phagocytosis of macrophages is involved in the occurrence and development of DM and its complications

The phagocytic capacity of macrophages mainly refers to phagocytosis and elimination of foreign or endogenous antigens (phagocytosis), dead or damaged cells in vivo (efferocytosis), and digestion of materials absorbed by macrophages (autophagy). These abilities play important roles in both the innate and adaptive immune systems. There are pattern recognition receptors on the surface of macrophages that recognize the receptor targets “PAMPs” on pathogens.^[23]^ Specific receptors TIM-4, BAI1, and Mer tyrosine receptor kinase (MerTK) recognize exposed receptor targets on apoptotic cells “DAMPs (such as phosphatidylserine (PtdSer),^[24]^” Phagocytosis is initiated after activation of signaling pathways within macrophages. Phagocytosis is the primary function of macrophages - plasmacytomembrane invagination (remodeling of the actin cytoskeleton to form a “phagocytic cup,” with progressively extended pseudopodia to surround the target particle) followed by encapsulation of foreign particles in vesicles (the “phagocytic cup” closes its distal end to form a “phagosome”), which detaches from the cell membrane and enters the cytoplasm.^[25]^ After shedding from the cell membrane, the fusion of the vesicle with the lysosome results in the formation of a larger, acidified vesicle called the “phagolysosome.”^[26,27]^ As a professional phagocyte, macrophages promote tissue homeostasis by monitoring, maintenance, and repair, while also performing immune defense.^[28]^ Their phagocytic efficiency is higher than that of nonprofessional phagocytes, like fibroblasts, epithelial cells, and endothelial cells, enabling them to uptake and eliminate particles larger than 0.5 μm in diameter.^[26–29]^ Normally, these particles undergo the process of being recognized, phagocytized, transferred to lysosomes, and degraded within phagolysosomes, and eventually become small molecules such as amino acids, nucleotides, fatty acids, and monosaccharides.^[30,31]^ Upon release from lysosomes, these molecules can be recycled to generate new macromolecules. However, once the defect in phagocytosis or digestion of macrophages appears and is not promptly corrected, the immune system may become persistently activated, which may lead to the appearance of autoimmune diseases.^[30,31]^

One of the major sources of diabetes autoantigens is apoptotic beta cells. A number of studies have observed in the pancreas of non-obese diabetic mice (NOD) that macrophages cannot effectively phagocytose and eliminate apoptotic pancreatic β cells, which will continuously trigger and upgrade inflammatory responses, upgrade self-antigen presentation, and initiate autoimmunity to cause T1DM.^[32,33]^ Other studies have shown that coxsackievirus B (CBV) infection of pancreatic β cells does not directly cause β cell death, but infected β cells are phagocyted by macrophages and then presented to autoreactive T cells. These T cells direct their action to the islet tissue remaining during the infection and eventually induce T1DM.^[34]^

In the event of infection, macrophages serve as key members of the body’s defense system, killing and eliminating pathogens and infected cells, and repairing cells and tissues damaged by infection. Phagocytosis is an important manifestation of the body’s immune defense ability. In diabetic patients, a noteworthy observation has been made that the decreased phagocytic activity of macrophages exhibits a positive correlation with the risk of a variety of diabetic complications, especially in impaired wound healing and heightened susceptibility to pathogens. Impaired wound healing is a prevalent complication in patients with T1DM and T2DM. To initiate the subsequent phase of wound healing, a substantial number of neutrophils recruited to the wound site must be fully eliminated by macrophages.^[35]^ However, impaired efferocytosis of macrophages can delay the onset of the next phase of wound healing. In addition, a multitude of pathogens, including Salmonella typhimurium, Legionella pneumophila, and Mycobacterium tuberculosis, have developed mechanisms to endure and even propagate within macrophages^.[26–37]^ “The results of a cross-sectional study” by Restrepo et al suggest that macrophages from diabetic patients are less potent against Mycobacterium tuberculosis than, for example, obese patients and healthy individuals.^[38]^

Clinical trials using modified flow cytometry to assess the phagocytic activity of macrophages in peripheral blood have shown that the phagocytic activity of macrophages in peripheral blood is decreased in patients with T2DM,^[39]^ which may contribute to the heightened susceptibility to infection observed in diabetic patients. After the immune defense ability of patients with diabetes is reduced, the most susceptible pathogens are Mycobacterium tuberculosis, Staphylococcus aureus, Streptococcus pneumoniae, and Klebsiella pneumoniae.^[40,41]^ The reason for the higher susceptibility of monocytic phagocytes (including monocytes and macrophages) in T2DM patients to pathogens such as Mycobacterium tuberculosis may be related to the defective phagocytosis mediated by complement receptors (CRs) or Fc-γ receptors on the surface of macrophages.^[42]^ Diabetic patients infected with Burkholderia pseudomallei are more likely to show signs of septicemia.^[43]^ Hodgson et al collected peritoneal macrophages from the peritoneal cavity of mice with T2DM that were infected with Burkholderia pseudomallei. These macrophages were found to be significantly impaired in their ability to engulf and kill Burkholderia pseudomallei.^[44]^ This may make it difficult to control the escalation of infection in diabetic patients infected with Burkholderia pseudomallei and thus more likely to progress to sepsis.

Several lncrnas (e.g., lncRNA E330013P06, lncRNA Dnm3os) have been found to be upregulated by diabetes. It promotes the pro-atherogenic phenotype of macrophages (increased expression of scavenger receptor CD36), which leads to excessive phagocytosis of oxidized low-density lipoprotein (Ox-LDL) by macrophages, resulting in enhanced formation of foam cells, and an elevated risk of diabetic inflammatory vascular complications.^[45–47]^ Studies have shown that high glucose can activate the nuclear factor kappa-light-chain-enhancer of activated B cell (NF-κB) signaling pathway in macrophages to significantly increase the expression of lncRNA Dnm3os. In vitro, it was observed that lncRNA Dnm3os enhanced phagocytosis after interacting with nucleolin and ILF-2 in macrophages.^[46–48]^ High glucose and free fatty acids in diabetic mice can up-regulate the level of lncRNA E330013P06 in macrophages, and up-regulation of lncRNA E330013P06 can also activate CD36-dependent signaling cascade, increase foam cell formation, and increase atherogenic potential.^[45]^

## 4. Abnormal efferocytosis of macrophages is involved in the occurrence and development of DM and its complications

**Figure 1.**
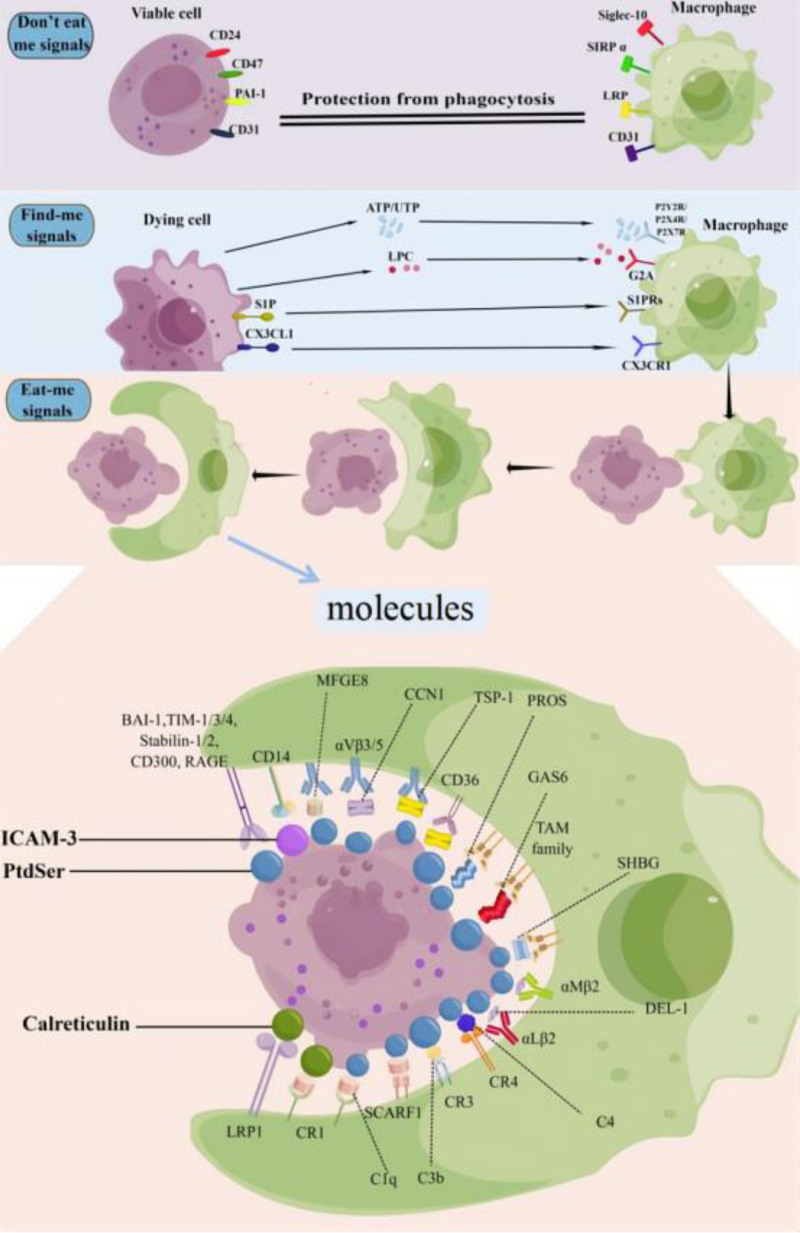
Macrophage efferocytosis process. This figure illustrates the multi-step process of efferocytosis, where macrophages identify, approach, and engulf dying cells to maintain tissue homeostasis. The process involves several key signals and receptors: Viable Cells: Emit “don’t eat me” signals (e.g., CD47) that prevent macrophages from mistakenly targeting them for clearance. Dying Cells: Release “find-me” signals (such as ATP/UTP and LPC) to attract macrophages and display “eat-me” signals (e.g., phosphatidylserine) on their surfaces. These signals are recognized by macrophage receptors directly or indirectly through “bridging” molecules. Signal Recognition and Phagocytosis: Upon detecting “eat-me” signals, macrophages initiate actin cytoskeleton reorganization to form a “phagocytic cup,” followed by the extension of pseudopodia to enclose the dying cell. Bridging molecules like MFGE8 and GAS6 assist in forming a stable “ligand-bridging molecule-receptor” structure, enhancing the phagocytosis process. Post-engulfment: Macrophages degrade engulfed cells in phagolysosomes, releasing digested material to contribute to immune homeostasis.

Efferocytosis is the process of phagocytosis of dead and damaged cells by macrophages and is a specialized function of macrophages,^[49]^ as shown in Figure 1. The characteristic process of efferocytosis is that dying cells release “find me” signal (dying cells release molecules to recruit phagocytes, such as nucleotides ATP/UTP, lysophosphatidylcholine (LPC), sphingosine-1-phosphate (S1P), and Fractalkine (i.e., CXC3CL1) and the “eat me” signal (dying cells expose molecules on their surfaces that are recognized by phagocytes, such as PtdSer, intercellular adhesion molecule 3(ICAM-3) and Calreticulin).^[50–52]^ The receptors (such as the P2Y2 receptor, P2X4R, P2X7R, G protein-coupled receptor (G2A), S1PRs (S1P-R1, S1P-R2, S1P-R3, S1P-R4, S1P-R5), CX3CR1, complement receptors (CR1, CR3, CR4)) on the surface of macrophages recognize the “find me” signal and migrate towards the dying cells,^[53–57]^ after which the phagocytic receptors((such as T-cell immunoglobulin mucin (TIM-1, TIM-3, TIM-4), Brain-specific angiogenesis inhibitor-1 (BAI-1) and Stabilin-1, Stabilin-2, LDL-receptor related protein1 (LRP1), receptor for advanced glycation end products (RAGE), CD14 and CD300) directly recognize the “eat me” signal from the dying cells.^[58–61]^ The receptors (including TAM family (Tyro3, Axl, MerTK), αvβ3/5 integrins, CD36, scavenger receptor type F family member 1 (SCARF1), αMβ2 and αLβ2) on the surface of macrophages can also indirectly bind to apoptotic cells by contacting with bridging molecules(such as milk fat globule-EGF factor 8 (MFGE8), complement (C1q, C3b, C4), growth arrest-specific gene 6 protein (GAS6), protein S (PROS), developmental endothelial locus-1 (DEL)-1, CCN family member 1 (CCN1), and thrombospondin-1(TSP-1), sex hormone-binding globulin (SHBG)), completing the phagocytosis process in the form of “ligand-bridging molecule-receptor.”^[58,62–68]^ In addition, it’s important to note that there are “don’t eat me” signaling molecules (CD47, CD31, CD24, plasminogen activator inhibitor-1 (PAI-1)) on the surface of Viable cells that bind to receptors (SIRP α, CD31, Siglec-10)on the surface of macrophages to avoid being accidentally swallowed by the macrophages.^[69–73]^ The molecules involved in the efferocytosis process are shown in Table 1 and Figure 1. Afterward, the macrophages undergo 3 processes similar to the recognition of PAMPs on pathogens upon contact with dying cells: cytoskeleton recombination to promote the formation of “phagocytic cups,”^[74]^ formation of “phagocytic lysosomes” to digest and degrade “foreign substances,” and release of degraded small molecules from lysosomes.

**Table 1:** Efferocytosis-related signaling molecules

Role	Molecule	Expression	(Ligand-Bridging molecule-) receptor
Find-me signals	ATP/UTP	Dying cell	P2Y2R, P2X4R, P2X7R
	LPC	Dying cell	G2A
	S1P	Dying cell	S1PRs
	CX3CL1	Dying cell	CX3CR1
Eat-me signals	PtdSer	Dying cell	BAI-1, TIM-1, TIM-3, TIM-4, Stabilin-1, Stabilin-2, CD300, RAGE
	Calreticulin	Dying cell	LRP1
	ICAM-3	Dying cell	CD14
Bridging molecules	MFGE8	Dying cell	(PtdSer-MFGE8-)αVβ3 integrin, (PtdSer-MFGE8-)αVβ5 integrin
	PROS	Macrophage	(PtdSer-PROS-)TAM family
	C1q	Macrophage	(PtdSer-C1q-)SCARF1, (PtdSer-C1q-)CR1, (Calreticulin-C1q-)CR1
	C3b	Macrophage	(PtdSer-C3b-)CR3
	C4	Macrophage	(PtdSer-C4 -)CR4
	GAS6	Dying cell/Macrophage	(PtdSer-GAS6-)TAM family
	SHBG	Viable cell	(PtdSer-SHBG-)TAM family
	TSP-1	Dying cell	(PtdSer-TSP-1-)CD36, (PtdSer-TSP-1-)αvβ3integrin, (PtdSer-TSP-1-)αvβ5integrin
	DEL-1	Viable cell	(PtdSer-DEL-1-)αMβ2integrins, (PtdSer-DEL-1-)αLβ2integrins
	CCN1	Viable cell	(PtdSer-CCN1-)αvβ3integrin, (PtdSer-CCN1-)αvβ5integrin
Don’t eat me signals	CD24	Viable cell	Siglec-10
	CD47	Viable cell	SIRP α
	PAI-1	Viable cell	LRP
	CD31	Viable cell	CD31

### 4.1. Signaling pathways associated with macrophage efferocytosis

Endocytosis-related molecules can effectively regulate macrophage efferocytosis and play a crucial role in the process of resolving inflammation and promoting tissue repair. DEL-1 can improve macrophage efferocytosis and inhibit the inflammatory response. Treatment with DEL-1 can up-regulate SIRT1 expression in C2C12 muscle cells and skeletal muscle of diabetic mice, as well as to increase the expression of SERCA2 in a dose-dependent manner^[75]^ to improve insulin resistance^[76,77]^and endoplasmic reticulum (ER) stress-induced inflammation (ER stress can trigger NF-κB activation via NOD1 and/or NOD2 signaling^[78]^ and promote M1 polarization in macrophages^[79]^). In addition, it has also been shown that expression of endogenous apoE4 in macrophages impairs macrophage efferocytosis by activating endoplasmic reticulum stress signaling pathways (e.g., IRE-1α/XBP1 pathway, PERK/ATF4 pathway).^[80]^

Similarly, the signaling pathways that affect the expression of the above-mentioned endocytosis-related molecules will inevitably affect the efferocytosis of macrophages. Most studies suggest that Sirtuin 6 (SIRT6) has a positive role in maintaining insulin sensitivity and glucose homeostasis, and is involved in regulating blood glucose homeostasis in vivo.^[81]^ However, SIRT6 is actually under-expressed in the tissues of many diabetic patients.^[82,83]^ When high glucose down-regulates the expression of SIRT6 in mice, the inhibitory effect on MIR217HG transcription is weakened. Once the 3 miRNAs encoded by MIR217HG (miR-216a, miR-216b, and miR-217) mature,^[84]^ they can target and impede the expression of DEL-1 and CD36. Macrophage efferocytosis within tissues is obstructed, leading to an increase in the accumulation of apoptotic neutrophils, and the balance of M1/M2 polarization is disrupted, which ultimately leads to protracted regression of periodontal tissue inflammation.^[85]^ Moreover, Lnc OIP5-AS1 and miR-137 have also been discerned to regulate the expression of DEL-1 to improve macrophage efferocytosis, which can reduce periodontal inflammation and accelerate the regression of inflammation in diabetic mice.^[86]^

In addition to the impact on macrophages efferocytosis exerted by endocytosis-related molecules, the imbalance between Ras-related C3 botulinum toxin substrate 1 (Rac1) and RhoA during the formation of phagocytic cups also hinders the reorganization of the actin cytoskeleton within macrophages. This interference (with Rac1 positively modulating the phagocytosis rate of apoptotic cells and RhoA negatively regulating it), ultimately culminates in the inhibition of efferocytosis.^[87]^ The continuous high glucose status leads to elevated levels of advanced glycation end products (AGEs),^[88]^ which bind to the RAGE of macrophages and activate RhoA/ROCK signaling pathway (ROCK is a downstream target effector molecule of RhoA^[89]^; when RhoA is activated, it can stimulate FilGAP via ROCK, thereby inhibiting Rac1 activity^[90,91]^), inhibiting the phagocytosis ability of macrophages by inhibiting Rac1 activity and cytoskeletal rearrangement.^[92]^ In addition, when glucose metabolism is disturbed, the increased AGEs in the body can competitively inhibit the binding of the endocytosis-related molecule PtdSer to RAGE, thus further inhibiting efferocytosis.

Plasma miR-126 is a potential biomarker for diabetes mellitus (DM), with decreased levels increasing the risk of cardiovascular complications.^[93]^ Comparing left ventricular free wall tissue samples from healthy individuals and diabetic patients reveals lower miR-126 levels and higher ADAM9 levels in the latter.^[94]^ Elevated glucose levels can decrease the expression of miR-126 in macrophages, resulting in increased expression of its target gene, ADAM9^[95,96]^ ADAM9 can cleave MERTK on the macrophage surface and inactivate downstream efferocytosis-related signals,^[97]^ resulting in efferocytosis defects of macrophages (impaired ability to phagocytose and clear apoptotic cardiomyocytes (ACM)) and aggravating inflammatory responses in the heart. Conversely, effective regulation of the miR-126/ADAM9/MERTK signaling axis may rescue high-glucose-induced efferocytosis dysfunction and alleviate inflammation after tissue injury,^[94]^ which has the potential to promote the potential of cardiac repair after injury.

### 4.2. Effect of dysfunctional macrophage efferocytosis on DM and its complications

Efferocytosis disorder not only affects the processing and recycling of waste but also exhibits a strong correlation with the persistence of inflammation.^[98]^ When efferocytosis is normal, macrophages can inhibit the release of pro-inflammatory cytokines (TNF-α, IL-1β, IL-6) stimulated by LPS, and promote the secretion of pro-inflammatory cytokines (IL-10 and TGF-β).^[92]^ When efferocytosis is compromised, the uncleared apoptotic cells transform into necrotic cells, and these secondary necrotic cells can release pro-inflammatory cytokines (such as TNF-α, IL-8, IL-1, and IL-6) to exacerbate the inflammation in the body, which is not conducive to the maintenance of tissue homeostasis.^[99]^ Therefore, efferocytosis plays a pivotal role in regulating the body’s inflammatory reactions and promoting the resolution of inflammation. In the pathogenesis of diabetes, insufficient clearance of apoptotic cells from the pancreas by macrophages may promote autoimmunity and cause damage to islet β cells by activating inflammatory signals.^[100,101]^

Chronic tissue inflammation is a defining characteristic of the pathogenesis of diabetic complications.^[102]^ In gingival tissue biopsies from patients with periodontitis and diabetes, the polarization of macrophages (M2/M1 ratio was significantly lower than that of periodontitis patients without diabetes) was dysregulated, and the impaired macrophages efferocytosis led to excessive accumulation and delayed clearance of apoptotic neutrophils and neutrophil extracellular traps (NET). This could potentially contribute to the persistent inflammation observed in diabetic patients who suffer from periodontitis.^[85]^ Furthermore, Yamashita et al used a streptozotocin-induced model of T1DM to investigate factors affecting lung tissue repair after lung injury. The study revealed that the myeloperoxidase (MPO) activity assay did not detect any difference in the number of macrophages engulfing apoptotic neutrophils between β-cell-depleted mice and non-diabetic mice. However, the inability of alveolar macrophages engulfing apoptotic neutrophils to induce higher levels of HGF production (one source of HGF is alveolar macrophages after engulfing neutrophils^[103]^) also means that the process of healing and repair of damaged lung epithelium will be disturbed.^[104,105]^ After intratracheal administration of LPS, β-cell-depleted mice have prolonged resolution of lung inflammation,^[103]^ which means that patients with T1DM complicated with pneumonia are more likely to develop chronic pneumonia due to impaired lung tissue repair due to impaired alveolar macrophage efferocytosis.

In bone tissue, macrophages are referred to as “osteoclasts”^[106]^ and play a crucial role in bone resorption, making them one of the target cells in the treatment of osteoporosis. Loss of bone mineral density (BMD) and osteoporosis are due to excessive bone resorption by osteoclasts, coupled with diminished bone formation by osteoblasts.^[107]^ Both in vitro and in vitro experiments, it was found that high glucose concentrations could induce reactive oxygen species production in osteoclasts, activating the MAPKs/NF-κB/NLRP3 inflammatory pathways. Concurrently, impaired efferocytosis in osteoclasts hinders the timely clearance of apoptotic neutrophils, exacerbating inflammation. This leads to the enhancement of bone resorption capacity of osteoclasts, and finally disrupts the osteoclasts-osteoblast balance in the process of bone remodeling and promotes bone destruction.^[108,109]^

After conducting clinical research, it has been observed that the level of MFG-E8 in the wound fluid of diabetic patients is significantly reduced, and under high glucose conditions, MFG-E8 is glycosylated, leading to the loss of its binding activity with phosphatidylserine (PtdSer), which further attenuates efferocytosis.^[110]^ As a result, macrophages within the wound are unable to eliminate apoptotic cells in time, which impedes the resolution of inflammation and hinders the progression of wound healing to subsequent stages.^[35]^

## 5. Effect of dysfunctional macrophage autophagy on complications of DM

Autophagy and phagocytosis are used to capture and digest the internal and external substances of the cell, respectively. In the process, phagosomes are usually single-layer vesicles with Fc receptors (FcR) and complement receptors (CR) on the membrane surface,^[111]^ and autophagosomes are usually double-membrane vesicles with proteins such as LC3 and p62 on the membrane surface.^[112]^ Both of them can fuse with lysosomes to form different degrading-vesicles (autolysosome and phagolysosome) to provide nutrients and energy for cell survival.^[26]^ Eukaryotic cells employ 3 principal intracellular pathways of autophagy, namely macroautophagy, microautophagy, and chaperone-mediated autophagy, distinguished by their unique physiological functions and mode of delivery to the lysosomal lumen. But they share the common fate of lysosomal degradation.^[113]^ Macroautophagy is the most widely studied of the 3 forms of autophagy. In this context, we will specifically concentrate on the mechanism and significance of macroautophagy (henceforth referred to as “autophagy”) in the pathogenesis of diabetes and its associated complications.

Autophagy begins with the formation of autophagosome- double-membrane vesicles that wrap the cellular components that need to be decomposed into autophagosomes, and is transported to lysosomes for decomposition into autophagic vacuole. This is typically a process of degradation of the cell’s own cytoplasmic proteins and damaged organelles.^[114]^ β-cells autophagy is a process in which β-cells recycle their own excess or damaged cell components in order to maintain the stability of the internal environment and survive under stress (mainly regulating the systemic insulin level in response to blood glucose level), and it is also an adaptive response to maintain the normal function and survival of β-cells.^[115]^ Thus, the role of impaired β-cells autophagy in the pathogenesis of T1DM and T2DM is intuitive.^[116–119]^ β-cells autophagy mainly degrades intracellular components,^[115]^ while macrophages can phagocytose extracellular components for degradation, such as fat, silica, etc.^[120]^ Consequently, we recognized the crucial role of macrophage autophagy in the formation and development of diabetic complications. As previously mentioned, diabetes increases the risk of microvascular and macrovascular complications.^[121,122]^ The malignant remodeling of atherosclerotic plaques in diabetes mellitus is related to the migration of foam cells into the lesion of arterial wall (lipid autophagy disorder occurs in macrophages recruited in the intravascular subcutaneous space, resulting in lipid accumulation in macrophages) and the imbalance of mononuclear macrophages migration efflux.^[123]^ Following an elevation in blood glucose levels, the concentration of glycation substrates increases, leading to a rise in the content of AGEs within the bloodstream.^[124]^ The primary active component of, Nε-carboxymethyl-Lysine (CML), upregulates the expression of macrophage scavenger receptor CD36, promoting intracellular lipid accumulation and attenuating foam cell migration capacity.^[125,126]^ Ultimately, arterial wall plaques become the final destination for non-migrating foam cells. Additionally, wound healing dysfunction, commonly observed in diabetic patients, is not only related to macrophage polarization imbalance but also closely associated with heightened macrophage autophagy. Upon administering AGEs to wounds in diabetic mice, an excessive augmentation of macrophage autophagy activity is observed (marked increase in the number of autolysosomes and elevated levels of LC3 protein), resulting in delayed wound healing.^[126]^

## 6. Therapeutics targeting macrophages in DM and its complications

Currently, research on the role and mechanisms of macrophage phagocytic ability in diabetes and its complications is continually advancing, providing a wealth of theoretical support for future prevention and treatment strategies targeting macrophages in diabetes and its complications. Many studies have also demonstrated the potential for targeted regulation of macrophage functions. Although blood glucose control remains the primary therapeutic approach for diabetes, it is not entirely effective in preventing or reversing the multitude of complications faced by diabetic patients. To address these complications, it is imperative to adopt therapeutic strategies targeting the underlying mechanisms.

### 6.1. Regulating macrophage phagocytic capacity to reduce DM-related vascular disease risk

High glucose concentration can upregulate Sterol Regulatory Element Binding Protein 1 (SREBP1) expression and maturation, leading to increased macrophage uptake of Ox-LDL, enhanced cholesterol biosynthesis, reduced cholesterol efflux, and cellular lipid peroxidation, resulting in an increased formation of foam cells.^[127]^ Insulin, on the other hand, can suppress SREBP-1 activation, transcriptional expression of HMG-CoA reductase (reducing cholesterol biosynthesis), inhibit CD36 expression (reducing Ox-LDL uptake), and suppress NADPH oxidase expression(avoid lipid peroxidation), thus reducing macrophage foam cell formation and atherosclerosis incidence.^[128]^ Additionally, the antidiabetic drug Dipeptidyl peptidase-4 (DPP-4) inhibitor, sitagliptin, can downregulate CD36 and cholesterol acyltransferase-1 (ACAT-1) expression, decreasing lipid accumulation in macrophages, offering vascular protection for T2DM patients.^[129]^ Research has also observed that vitamin D deficiency may reduce macrophage autophagy-mediated lipid breakdown.^[130,131]^ Upon vitamin D supplementation, the 1,25(OH)2D3 signaling pathway can alleviate diabetic patients’ susceptibility to atherosclerosis through several mechanisms: mitigating endoplasmic reticulum stress in macrophages, improving insulin signal transduction, and downregulating scavenger receptor SR-A1 expression; as well as downregulating c-Jun N-terminal kinase activation, which in turn decreases PPARγ and scavenger receptor CD-36 expression.^[132–134]^ Ultimately, due to the reduced expression of macrophage surface scavenger receptors CD-36 and SR-A1, the uptake of acetylated-LDL (AcLDL) and Ox-LDL cholesterol is diminished, decreasing foam cell formation and the risk of vascular complications in diabetic patients. Intermedin (IMD), a calcitonin peptide, can inhibit NF-κB signaling^[135]^ and upregulate miR-3b-7p expression to suppress Dnm27os and SLAMF3, thereby inhibiting macrophage phagocytic activity, avoiding excessive Ox-LDL uptake, and reducing atherosclerosis formation in diabetic mice.^[48]^

Fish oil diets, rich in n-3 FAs (Eicosapentaenoic acid (EPA) and Docosahexaenoic acid (DHA)), can elevate PI3K activation levels in macrophages of T2DM mice, thereby enhancing the uptake and digestion of apoptotic cells, and mitigating necrotic core formation and inflammation in atherosclerotic lesions. This dietary approach may help prevent atherosclerotic cardiovascular disease in T2DM patients by reversing macrophage efferocytosis functional deficits.^[136]^

### 6.2. Regulating macrophage phagocytic capacity to reduce DM-related non-vascular disease risk

The endocytosis-related molecules MFG-E8 not only improve efferocytosis in macrophages at diabetic wound sites but also has anti-inflammatory^[137,138]^ and pro-angiogenic effects.^[137–139]^ These beneficial effects may simplify and coordinate the complex pathogenic factors (such as chronic inflammation and poor perfusion) that need to be addressed in the clinical management of diabetic wound complications, highlighting the extraordinary significance and potential of MFG-E8 targeted therapy in diabetic wound care.

In diabetic rats accompanied by adrenal cortex insufficiency, there is an increased production of lipoperoxides and heightened NOS activity in the adrenal cortex tissue. Through antioxidant treatments, such as alpha-lipoic acid or alpha-tocopherol, oxidative stress in the adrenal cortex can be alleviated, preventing excessive cellular apoptosis and overactive macrophage phagocytosis, thus averting HPA axis dysfunction.^[140,141]^

The efferocytosis enhancer LXA 4 treatment can slow down the development of diabetic osteoporosis by increasing the efferocytosis of osteoclasts in the body, and can also reduce the expression of inflammation-related pathway proteins MAPKs, NF-κB, and NLRP3 inflammasome-associated proteins, which may serve as a potential drug for the treatment of diabetic osteoporosis.^[141]^

In diabetic mice, the phagocytic capacity of hepatic macrophages, known as Kupffer cells, declines, leading to an upregulation of pro-inflammatory cytokines (TNF-α, IL-6, and IFN-γ) and increased expression of ICAM-1 in Kupffer cells, resulting in enhanced recruitment of granulocytes. Lidocaine, however, can reverse these aberrant macrophage functions in diabetic mice, thereby exerting a beneficial effect on the progression of diabetes and the occurrence of complications.^[142]^

## 7. Conclusion

Overall, inflammation in peripheral tissues is a key factor in the progression of diabetes, and a comprehensive understanding of macrophage phagocytic function is crucial for exploring the pathogenesis of diabetes and its complications, as well as the “damage-response” mechanisms. However, significant challenges remain in translating macrophage research into effective clinical applications. These challenges include the lack of therapies directly protecting pancreatic β-cells with macrophages as the entry point, which is critical for preventing autoimmune damage and preserving β-cell function, the limited clinical trials for macrophage-targeted therapies, despite their promise in preclinical studies, and the complex network of molecules influencing macrophage function and activation state, where imbalances can disrupt macrophage-mediated homeostasis. Currently, processes such as macrophage phagocytosis, efferocytosis, and autophagy are often studied independently within simplified contexts to assess their roles in diabetes and its complications. Nevertheless, the progression of diabetes involves intricate biological mechanisms that require a more integrated approach. Thus, future research should deepen understanding of macrophage plasticity and its regulation, with a focus on modulating key functions like phagocytosis and anti-inflammatory responses to create effective, clinically applicable therapies.

A thorough comprehension of macrophages’ pivotal role in immune responses will aid the seamless transition of fundamental research into clinical practice, providing a foundation for novel macrophage-targeted therapies designed to mitigate diabetes complications.^[143]^ As demonstrated by recent findings,^[144,145]^ macrophages exhibit remarkable plasticity, supporting the future development of innovative therapeutic strategies aimed at reducing the risk of diabetes and its associated complications.

## Acknowledgments

We are grateful to Figdraw for helping us produce figures.

## Author contributions

**Conceptualization:** Bing Rong, Hailun Jiang.

**Investigation:** Guanhu Yang, Xuancheng Zhou, Zhongxi Lyu, Xiangyi Li, Jieying Zhang.

**Project administration:** Jieying Zhang.

**Supervision:** Jieying Zhang.

**Writing – original draft:** Bing Rong, Hailun Jiang.

**Writing – review & editing:** Weiming Zhu, Jieying Zhang.
